# On the Development and Structure of the Teeth

**Published:** 1841

**Authors:** 


### ON THE DEVELOPMENT AND STRUCTURE OF THE TEETH.

(From the Boston Medical and Surgical Journal, for September, 1839.)

Investigations respecting the structure and development of the teeth have been carried on during the last few years, by some of the most celebrated anatomists now living; and it is with much satisfaction that we are enabled to place before our readers the highly interesting and valuable results at which they have arrived. In the present article we propose to examine, first, the structure, and secondly, the development, of the teeth.

I. The Structure of the Teeth.—In a paper published in the Philosophical Transactions for the year 1678, Leeuwenhoeck announced that, having extracted one of his own teeth, he examined it with lenses, and that he and others "plainly saw that the whole tooth was made up of very small, straight, transparent pipes; of these pipes Leeuwenhoeck gave two figures, and he spoke of their existence in the teeth of the cow and of the haddock. In the year 1687, the same author wrote a continuation of the above researches, in which he further explained the nature of these "pipes," as existing in man and various animals; and he described a molar tooth of the human subject, which was the object of very careful investigation, to contain 4,822,500 pipes or tubes. This discovery of Leeuwenhoeck remained unnoticed during many years.—Writers upon the structure of the teeth who succeeded him, and they have been numerous, contented themselves with describing the appearances observed upon examination by means of the naked eye. Purkinje and Retzius, unknown to each other, conducted researches upon the structure of the teeth, with the assistance of the microscope.

In the year 1835, the former, through his pupil Frankel, announced the discovery of the tubular structure of ivory. Nearly at the same time, Retzius, in a series of letters to the Royal Society of Stockholm, published the details of his researches. Muller, in his Archives for 1835, analyzed the above researches, and made important additions to them. Mr. Owen has conducted his extensive survey of the same subject into the field of comparative anatomy and geology. Mr. Tomes, another of our countrymen, has also written an original and very interesting paper upon this subject. It is at once a proof of the accuracy of the above observers, and of the perfection which the microscope has attained, that although the use of the highest compound lenses has been found requisite, in order to reveal the more minute structures, all the observers, whose names we have mentioned, agree in every point. We hope that the knowledge of this fact will prove a satisfactory answer to those who refuse to use the microscope, and to listen to the results derived from its use, "because it is liable to so many fallacies."

After the perusal of Brewster's paper "On the Structure of the Crystalline Lens," published in the Philosophical Transactions for the year

1833, which proved that the peculiar mother-of-pearl color of that structure depends upon its fibres ; it occurred to Retzius, that the same peculiar color possessed by dental bones might also depend upon the entering into its composition of minute fibres. These minute fibres Retzius readily discovered ; and he afterwards proceeded to conduct researches into the structure of the teeth generally.

1. *The Ivory or Bone of the Tooth.*—Retzius states that " having made a fine section of a tooth that had been placed in diluted muriatic acid, and having examined it by means of transmitted light, with a lens magnifying as low as 60 diameters, it will be quite apparent to the observer that it is composed of undulating fibres, in apposition internally with the *cavitas pulpæ*, and externally with the surface of the tooth."—If an oblique section be afterwards made, and a magnifying power of 200 diameters be employed, these fibres will appear to be hollow. Retzius found that the above fibres or tubes gave off branches, and that the main trunks opened into the cavitas pulpæ. Although these branches are principally evident towards the external surface of the tooth, the canals at their origin may be distinctly seen to present dichotomous divisions.

Retzius details the appearances revealed by the microscope in the teeth of man and the lower animals. In man, " the tubes which are contiguous appear to run parallel ; upon examining a number, they are found to radiate from the central cavity towards the circumference." These tubes have, more or less, three curves, resembling the Greek letter ξ.—These curves vary in different teeth, approaching sometimes in form to the Roman letter S. In well-formed teeth, the curves on each side present a certain symmetry. Independent of these general curves of the entire tube, under a higher magnifying power are observed " numerous shorter curvatures, nearly 200 of which may be counted in the space of a line ;" these produce the appearance in each tube of an undulating line. Professor Owen has remarked partial dilatations in the tubes of the human teeth, an appearance which has not unfrequently been noticed by ourselves.

Leeuwenhoeck endeavoured to ascertain how it was possible that tubes, which appear to run parallel to each other, should fill such a different space on the surface of the tooth, and at the internal cavity. He sought in vain for a trace of branches from them. Purkinje discovered, however, that these tubes do give off branches. Retzius says of them, " from their commencement in the cavitas pulpæ, the tubes appear to be of the same diameter for five sixths of their course;" and this diameter, after repeated admeasurements, he states to be $\frac{1}{385}$ of a line. After running five sixths of their course, they are found to diminish considerably in size until they disappear or terminate in small, irregular, rounded, and scattered cells. The branches of these tubes are more easily observable in milk teeth. In the permanent teeth, the branches are formed principally towards the extremities of the tubes ; in the milk teeth they arise nearly equally from all parts. The branches arising from different tubes do not communicate with each other, except perhaps at their extremities.

*Are these fibres tubular?*—Retzius states that the cavitas pulpæ, viewed under a sufficiently high magnifying power, presents numerous orifices which he considers to be the mouths of the main tubes. He also states that, by making sections of the tubes at right angles with their course, the calibre of these tubes may be determined with facility; and that, upon placing a section made in the above manner upon a dark ground, white spots distinctly denote the tubular orifices. Purkinje and Frankel convinced themselves of the tubular nature of these fibres, from finding, on making various sections of the tooth, that they were always able to demonstrate the parietes and cavity of the cut tube. Muller states that he has seen these fibres or tubes in the horse, injected with red coloring matter. Mr. Tomes has made some conclusive experiments upon this subject. After having reduced a transverse section of a giraffe's tooth, so as to render the tubes visible by the aid of transmitted light, he added to it, while under the microscope, diluted muriatic acid. "Chemical action immediately commenced, gas was disengaged and proceeded from the cut extremities of the tubes." He states that, more than once, he saw "the bubbles of gas in the tubes, and traced them to their extremities from which they escaped."

*The Parietes of the Tubes.*—Muller has made some researches upon this subject, which are of much interest. He states that the diameter of the tubes is only one fifth or one sixth of the spaces between them; the structure in the intervening spaces necessarily forming the greater part of the bulk of the tooth. He says that, upon breaking fine sections of teeth perpendicular to their fibres, he has frequently seen the latter "extend from the margin beyond the tooth-substance, seeming to be perfectly straight and inflexible;" but the earth being removed from slices of teeth, by means of acids, and the latter being torn in a direction contrary to the course of the fibres, "these fibres appear, upon the torn margin, quite flexible and transparent, and they not unfrequently project considerably beyond the margin of the section." Muller thinks that it may be inferred, from the above experiment, that the tubes have an animal basis or membrane, which in the firm tooth is fragile, and probably penetrated by calcareous salts. Upon this subject Retzius says that, under a certain light, "the mouths of the canals appear bounded by a definite shadow;" and their walls, when the light falls upon them, have a different appearance from the surrounding matter, which Frankel calls the fundamental substance of the tooth. Frequently the circular orifices have a darker and somewhat yellowish appearance. "We may conclude," says Retzius, "from all this, that the above-described tubes are not merely hollowed out of the fundamental dental substance, but that they are tubes properly so called, consisting of a particular substance, differing from that of the tooth."

*The Contents of the Tubes.*—Muller was the first who made investigations upon this branch of the subject. He considers the tubes to be filled with organic deposits of calcareous salts, which are soluble in acids. According to him the white color of the tooth is dependent upon these contents, the intervening substance being more or less transparent. He says, "The white color of the tooth disappears upon the application of acids; and teeth thus treated do not regain their colour when dried." Observers agree that even if the enamel only be carious, or rather the ivory immediately internal to the enamel, then the tubes passing towards the cavitas pulpæ lose their white color. Muller states that he has seen in carious teeth, independently of the transparent appearance above alluded to, a brittle substance contained in different parts of the tubes; he has, however, also observed the latter in perfectly healthy teeth, and he also speaks of the existence of opaque spots in the course of the tubes. According to Retzius, the contents of the canals consist of an inorganic or earthy substance, which appears white when seen on a dark ground. It appears to consist of "small masses, formed of infinitely delicate particles." The greater or less number and the degree of visibility of these particles appear to depend upon the extent to which the preparation has been penetrated by water, oil, or turpentine. Mr. Tomes agrees with Retzius as to the contents of these tubes. In mammalia he states them to consist of an amorphous mass, the composition of which is the phosphate and carbonate of lime. He concludes that the latter enters into the formation of the above mass, from the results of the experiments which we have before noticed, where he placed a thin section of the tooth of the giraffe in muriatic acid, and observed under the microscope the evolution of gas from the interior of the tubes. Mr. T. states that these tubes in the cartilaginous and osseous fish contain much less of the earthy matter than do those of animals higher in the scale.

*The Use of the Tubes.*—Retzius believes that the tubes of the teeth, as well as the canals of bone (canals of Havers), are for the circulation of nutritious fluids, which he imagines to be secreted by the capillaries which clothe the surface of the pulp. We quote from Retzius the following interesting observations upon this subject.

"We have many examples," he says, "that Nature organizes structures which have a close affinity to each other, according to one and the same plan, and hence we have, in different parts, organized formations, which in some are of the greatest importance, whilst in others they are of much less functional significance, or of none whatever. When we assume, what is highly probable, that in bone the peculiar vessels in question give passage to fluids during the entire life of the animal, which fluids contain the solid as well as the liquid materials of the osseous substance, it does not necessarily follow that the same process must be carried on in the teeth during the whole of life; on the contrary, I am inclined to believe that the vessels of dental bone exercise their most perfect action during the first period only of the formation of the tooth. At the same time, the existence of the continual vital process in the tooth, as well as in the crystalline lens, cannot be doubted, which appears, however, to be carried on without any constant exchange of solid matter, and must hence consist in a renovating circulation of fluids."

Mr. Tomes "conceives that the tubes, containing as they do an amorphous substance, could, by capillary absorption, carry on a kind of slow circulation of the more fluid parts of the blood." Professor Owen accounts for the good effects produced by stopping decayed teeth, by "the calcareous salts in such cases pouring out from the extremities of the tubes divided in the operation; then a thin dense layer intervenes between the exposed surface of the ivory and the stopping."

*Of the Intertubular Substance.*—Independently of the salts of lime contained in the cavity of the tubes, and of those which, with the animal basis, form the walls of these tubes, Muller considers that salts of lime also enter into the composition of the intervening substance; and, as we have seen above, that the tubes themselves are only of a size equal to one fifth or one sixth of the space between them, the greater portion of calcareous salts which enter into the composition of the tooth must be contained in the intertubular spaces.—Muller considers the lime to be either chemically combined with the cartilage of the tooth, or to be deposited in it in an insoluble manner. The lime-earth of the intertubular spaces may be demonstrated by carefully boiling slices of teeth in potash during many hours. The cartilage is dissolved, and the intervening substance becomes opaque and white. According to Muller, the lime thus obtained appears in the form of dense grains. Between the tubes are observed also the *corpuscles or osseous cells of the teeth*, called by Professor Owen the *calcigerous cells.* Some of the smaller tubes terminate in these cells. Valentin believes them to be analogous to the corpuscles of bone.—Retzius states "that they, as well as the tubes, contain earthy salts." This he proves by causing their white color to disappear, by placing the preparation in diluted muriatic acid.

*The Terminations of the Tubes*, according to most observers, take place—first, by entering the corpuscle or cell; secondly, by forming plexuses with one another; thirdly, by being lost in the intertubular substance, either in the interior of the ivory or at its surface of attachment to the enamel and crusta petrosa; fourthly, by communicating with the cells of the crusta petrosa.

2. *The Enamel of the Tooth.*—This is also called the vitreous and adamantine substance. Berzelius states that enamel *does not contain any animal matter.* He considers that the delicate tissue which remains after its earthy matter has been dissolved in acid, is the residue of a membrane which formed the internal lining to it. He says that "enamel is not at all blackened on the outside, and but very slightly on the inside, by being burned; and that it does not lose more than two per cent. of its weight from combustion." Retzius, with Berzelius, agrees in the existence of a membrane which lines the enamel internally, and which is a band of connection between it and the external surface of the ivory. Retzius believes that the inorganic fibres of enamel are surrounded by a capsule of organic matter; a view which we shall see is adopted by Purkinje and Raschkow. The researches of Mr. Nasmyth* show the existence of a layer of substance external to the enamel, and intimately connected with it. This layer Mr. N. has demonstrated in the tooth of the human subject, in the calf, and in many other animals; and he considers its presence to be almost universal. It is shown by the immersion of the tooth in diluted muriatic acid during a very short space of time, when it can be removed in the form of a membrane. It is continuous with the layer called the *crusta petrosa* on the surface of the fang, to which it seems to bear the greatest analogy, and, like the latter, contains the characteristic cells or corpuscles. The existence of this layer on the surface of the enamel, as pointed out by Mr. Nasmyth, we have since demonstrated, by exposing teeth to a rather high temperature, when a *very thin layer of black substance is recognized on their external surface:* it is more easily perceived, and it is thicker in young than in old teeth. Retzius agrees with Purkinje and Raschkow, in believing that the enamel, previous to the eruption of the tooth, consists of delicate prisms; each prism, upon its solution in muriatic acid, leaves behind a small portion or organic matter. As the latter is not found at a later period, Retzius supposes that it is supplanted by the deposition of earthy matter, so as to remain in a quantity so small as to be incapable of demonstration. The enamel fibres are described by Retzius to be $\frac{1}{61}$ of a line in diameter, the surface of which is marked with transverse striæ, which Retzius believes to be produced by the above-mentioned organic sheath.—The fibres of the enamel internally rest upon a delicate membrane interposed between the latter and the ivory. Externally, these fibres present a hexagonial form, which is seen by the aid of a lens, magnifying 300 diameters. Where the tooth has not been worn away, the extremities of these fibres are rounded. The enamel fibres are supported upon the ivory in different directions. Towards the apex of the tooth the direction is vertical, and the lowest fibres are transverse. In the lateral part of the tooth there are fibres which are interposed between other fibres, and which themselves do not reach the surface of the ivory. Where the enamel joins the

* In a paper read at the Medico-Chirurgical Society.

dental bone, it contains at different parts a number of delicate crevices, which appear to have their origins in the separation of the fibres, and produce a dentated appearance, similar to that of the crystalline lens. Retzius states that this appearance is rendered more evident by the immersion of the enamel in a solution of caustic potash, a circumstance which strengthens his belief in the existence of an organic sheath to the enamel fibres. Independently of the transverse striæ on the surface of each enamel fibre, the enamel presents a lamellated appearance, analogous to that of a calculus; this is also evident in ivory generally, but more particularly in that of the elephant's tusk. The fibres of the enamel, like those of the ivory, are more or less tortuous in their course.

3. *The Crusta Petrosa.*—It is universally known that ivory and enamel form the principal part of all simple teeth; and previously to the recent investigations in odontology, it was supposed that no other substance was present in the latter. Retzius and Purkinje, have, however, proved that a third substance enters into the formation of almost *all teeth.* This substance is the *crusta petrosa* or *cortical substance.* Frederick Cuvier spoke of this structure as existing in the cachalot. It is found on the external surface of the fangs of the teeth of the mammalia. It consists of cartilage and of osseous earth. According to Retzius, "this cartilage may be removed from the fang of the tooth in the form of a membrane, its earthy salts having been previously dissolved in acid; its consistence is less than that of the cartilage of ivory. In the human tooth, it is an extremely thin stratum, which takes its origin at the neck of the tooth where the enamel terminates; it gradually increases in thickness towards the extremity of the root, where it is generally the thickest. It is thinner in young than in old persons. In proportion as the cavitas pulpæ diminishes, this substance is developed until it sometimes becomes thicker than the dental bone itself." The exostoses, so frequently met with on the fangs of the teeth, are of a composition similar to this structure. It is imperfectly formed in the deciduous teeth. Examined by means of the microscope, it presents tubes and osseous cells, bearing the greatest analogy to those of bone and ivory. Professors Retzius and Owen have seen direct communications between the fine branches of the ivory and the tubes and cells of this substance. Mr. Nasmyth states that it always exists on the surface of the enamel; and Purkinje and Frankel "once traced it a short way upon the surface of the enamel of the tooth of an old man." The two latter observers state that the crusta petrosa has a lamellated structure; they have found it lining the cavitas pulpæ, and have given it the name of *substantia ostoidea.* Retzius thinks that after the obliteration of the cavitas pulpæ, the crusta petrosa is the medium through which nutrient fluids are conveyed to the tooth.

*The Cementum.*—The structure of which we have just spoken is generally considered to exist upon the root or fang of the tooth only, and to terminate at the point where the enamel is secreted, viz., at the neck of the tooth. As has been stated above, Purkinje and Frankel, however, once saw this substance covering a small part of the enamel of the tooth of an old man, and Mr. Nasmyth describes it as existing upon the surface of the enamel in most quadrupeds; and he has exhibited specimens and drawings of this disposition in the simple and compound teeth of the human subject, and in the simple tooth of the calf, &c. The cement is a deposition on the surface of the enamel, found in the compound teeth of the herbivorous animals; it has been described by Hunter, Blake, and many other anatomists.—Cuvier, in his "Ossemens Fossiles," has given a very minute and interesting account of its development and structure in the grinder of the elephant. It is formed by the capsule, as appears to be the crusta petrosa; it possesses corpuscles and tubes, as does the crusta petrosa; in short, it appears to us that the cementum is simply the crusta petrosa existing on the surface of the enamel: not only, as has been hitherto supposed, in the compound teeth of herbivorous animals, but according to the views of Mr. Nasmyth, in the simple and compound teeth of mammiferous animals. Muller most strangely describes the structure under consideration "as a deposit from the salts of the saliva, and to be essentially the same as what is called tartar in the human subject."

II. The Development of the Teeth.—The researches in this branch of odontology which have lately been published, and to the examination of which we shall at present confine ourselves, have an interest not much inferior to those of which we have already spoken. Much, however, in this department remains to be done; and observers have as yet to test the accuracy of the novel views which we are about to relate.

We shall speak—1st, of the Development of the Ivory of the Tooth; 2dly, of the Enamel; 3dly, of the Crusta Petrosa.

*The Development of the Ivory of the Tooth.*—The ivory of bone is formed by an organ called the pulp of the tooth. This pulp is at an early stage of development, contained in a cavity called the follicle, from the base of which it grows, and to which it is attached by vessels and nerves. According to Arnold and Goodsir, this follicle is thus produced: at a very early period of development (between the fifth and seventh weeks) in the human subject, a groove is observed on the free margin of the aveolar arch; from the floor of this groove the pulps of the teeth grow; after a certain period, septa are formed, which separate the pulps from each other, and divide the groove into a series of follicles, which continue to have a communication with the mucous membrane of the mouth, until a much later period. Arnold has seen the orifices of these follicles quite distinct in a child at birth. The researches of Mr. Goodsir have been conducted on a very extensive scale, and he has published delineations of the development of the follicle in all its stages ; at the time that he conducted his dissections, he was not aware of the views of Arnold, which appeared in the Salzburg Med. Zeitung for 1831. Purkinje and Rashkow are quite opposed to the above view of the development of the follicle, as described by Arnold and Goodsir. They state that they "have looked with the greatest care, and have made researches in comparative anatomy, but have never discovered a trace either of the groove or of the orifice of the follicle." Rashkow states that "the membrane of the follicle consists of soft fibres mixed with much granular matter ; at the earlier periods of development, this follicle is not united with the gum ; its internal surface is smooth like a serous membrane."

*The Pulp or Dental Germ.*—The pulp is developed in the interior of the above-mentioned follicle, which when the latter assumes the name of *capsule*, Raschkow considers "the pulp to be a production of the interior of the capsule, inasmuch as the two are inseparably connected, their vessels and nerves having a common origin." He denies that the dental germ is a continuation of the dental nerve.—The following is a condensed account of the views of Raschkow on the pulp and its functions. This organ, at its earliest period, consists of globular granulations, in which are not observed either vessels or nerves ; afterwards the vessels appear and then the nerves. The granules of nervous matter, in the interior of the pulp, assume the form of nervous cords, after the vessels are completely formed. In no part of the body are the extremities of the nerves so well seen as in the pulp, when it is mature. The nerve upon entering the pulp divides into filaments, the latter into a pencil of rays, in which they terminate. These filaments are accompanied by a cellular web. In the interior of the pulp of the hare, sow and stag, stony concretions may be observed towards the apex. A membrane covers the dental pulp from its base to its apex ; in this membrane the formation of the dental substance always commences. This membrane Raschkow calls "membrana præformativa," and it is described by him as being very dense. Raschkow has observed upon the surface of this membrane, previously to the formation of the dental substance, certain elevated spots. These he considers to be most probably transformed into the undulating ridges on the surface of the dental substance, to which the enamel is attached. According to Raschkow and Purkinje, the dental substance is formed immediately under the preformative membrane, the latter assuming an almost stony hardness ; between the dental germ and this ossified membrane, a layer of dental fibres is deposited from without inwards, the parenchyma of the dental germ supplying the materials. It will be seen that the views of the above observers differ much from those of preceding writers. Hitherto it has been supposed that the membrane covering the surface of the pulp, and intimately adhering to it, is the secreting organ of the ivory. Observers have accounted for the peculiar waving course of the tubes in ivory, by supposing that the pulp undergoes certain periodic movements.

*Development of the Enamel.*—We have seen that the first stage in the development of the tooth consists in the formation of a follicle or capsule, from the base of which grows an organ—the pulp, the function of which is to secrete the ivory of the tooth. Contemporaneously with the appearance of the pulp is the formation of another structure, a growth from the internal layer of the capsule; it is the formative organ of the enamel. It appears directly opposite to the ivory pulp, was called by Hunter the "enamel pulp," and by Purkinje and Raschkow the "adamantine organ." The researches of the two last-mentioned authors are highly interesting. They state that the "adamantine organ appears as a globular nucleus, having a granular structure, projecting into the cavity of the capsule towards the pulp of the ivory;" and that it eventually is converted into the membrane which secretes the enamel in the following manner: "It throws off towards its internal surface a stratum of fibres, producing the appearance of a silky covering. These fibres are eventually converted into a membrane—the membrane of the enamel. It has no traces of vessels or nerves. If examined with the microscope it will be found to consist of hexagonal bodies of almost equal size.—Each of these fibres must be considered a *gland*, whose function is to secrete a single enamel fibre, corresponding with itself. Each *gland* simultaneously with the earliest formation of the dental substance, deposits the primitive part of each adamantine or enamel fibre, one upon another, so that every one of these fibres, when carefully examined by the microscope, displays the order of its parts arranged in strata in a transverse direction." The enamel is first deposited upon the hardened preformative membrane mentioned above, and the progress of its formation corresponds exactly to that of the development of the ivory. At an early age of the production of these enamel fibres, the organic substance is a lymph which enters between each individual fibre, and appears to soften its entire substance; this organic substance appears to be formed by the parenchyma of the adamantine membrane between the above-named *glands*." Purkinje and Raschkow suppose that this animal substance, by means of a chemico-organic process, enters into close connection with the earthy matter, and thus forms the animal basis of the enamel, which is evident, by means of the microscope, after the latter has been placed in acid. These observers account for the production of the peculiar waving fibres of the enamel by the occurrence of certain movements in the adamantine membrane. The adamantine membrane is permanent in the incisors of the rodentia. Previous to the formation of the adamantine organ, it is supposed that the rudiments of this structure exist in the form of a serous sac.

*Development of the Crusta Petrosa and Cementum.*—Upon this branch of the subject much remains to be done. No new facts have been adduced since the time of Cuvier. The crusta petrosa and cementum are formed subsequently to the completion of the ivory and enamel; but whether the organ which produces them is a modification of those which formed the latter, or one for their especial development, anatomists have not yet determined.

The result of the investigations above detailed, is that the structure of the tooth is very analogous to that of bone. We shall refrain from making any observations upon this analogy until we have laid before our readers a view of the recent discoveries upon the structure of the latter tissue. Although we possess so full an account of the internal composition of the teeth, we are very deficient in researches into the development of each individual part of the tooth, as well as of the organ generally; we have noticed above that Purkinje and Raschkow entertain opinions directly opposite to those of Arnold and Goodsir; in fact, we believe, that upon no subject is there a greater diversity of opinion than upon the nature and functions of the structures in connection with the development, growth and organization of the dental apparatus. In support of this assertion, we refer to the first part of the work by Mr. Nasmyth. The cause of the strange discrepancies of opinion therein detailed, appears to arise from the too partial examination of the subject before us, which has been conducted by various observers.

Mr. Nasmyth promises to prosecute an extended series of researches into every branch of odontology; in doing so we feel sure that he will reconcile many of the present conflicting opinions, and we hope to receive from his hands, what is much wanted in medical literature, a Systematic Treatise on the Development, Structure, and Diseases of the Teeth.

To conclude: to those who wish to pursue the subject which we have so briefly brought under notice, we recommend the first part of the work of Mr. Nasmyth, as containing an entire translation of the papers of Retzius, which are illustrated by many beautiful and original plates; also a complete view of the researches of those whose names we have introduced in the present article; and, lastly, a comprehensive historical survey of all works on odontology.—*Brit. and Foreign Med. Review.*

